# Characterization of a Cohort of Patients With LIG4 Deficiency Reveals the Founder Effect of p.R278L, Unique to the Chinese Population

**DOI:** 10.3389/fimmu.2021.695993

**Published:** 2021-09-24

**Authors:** Xianze Luo, Qing Liu, Jinqiu Jiang, Wenjing Tang, Yuan Ding, Lina Zhou, Jie Yu, Xuemei Tang, Yunfei An, Xiaodong Zhao

**Affiliations:** ^1^ National Clinical Research Center for Child Health and Disorders, Children’s Hospital of Chongqing Medical University, Chongqing, China; ^2^ Ministry of Education Key Laboratory of Child Development and Disorders, Children’s Hospital of Chongqing Medical University, Chongqing, China; ^3^ China International Science and Technology Cooperation Base of Child Development and Critical Disorders, Children’s Hospital of Chongqing Medical University, Chongqing, China; ^4^ Chongqing Key Laboratory of Child Infection and Immunity, Children’s Hospital of Chongqing Medical University, Chongqing, China; ^5^ Department of Rheumatism and Immunology, Children’s Hospital of Chongqing Medical University, Chongqing, China; ^6^ Department of Healthy Examination Center, Children’s Hospital of Chongqing Medical University, Chongqing, China; ^7^ Department of Hematological Oncology, Children’s Hospital of Chongqing Medical University, Chongqing, China

**Keywords:** LIG4 deficiency, primary immunodeficiency disease, founder effect, haplotypes, mutation

## Abstract

DNA ligase IV (LIG4) deficiency is an extremely rare autosomal recessive primary immunodeficiency disease caused by mutations in LIG4. Patients suffer from a broad spectrum of clinical problems, including microcephaly, growth retardation, developmental delay, dysmorphic facial features, combined immunodeficiency, and a predisposition to autoimmune diseases and malignancy. In this study, the clinical, molecular, and immunological characteristics of 15 Chinese patients with LIG4 deficiency are summarized in detail. p.R278L (c.833G>T) is a unique mutation site present in the majority of Chinese cases. We conducted pedigree and haplotype analyses to examine the founder effect of this mutation site in China. This suggests that implementation of protocols for genetic diagnosis and for genetic counseling of affected pedigrees is essential. Also, the search might help determine the migration pathways of populations with Asian ancestry.

## 1 Introduction

DNA double-strand breaks (DSBs) are a deleterious form of DNA damage that can result in loss or rearrangement of genomic material, both of which lead to cell death or carcinogenesis (1–3). DSBs are induced by ionizing radiation, but they also arise as intermediates during normal endogenous processes such as DNA replication, and meiotic and V(D)J recombination. In mammalian cells, non-homologous DNA end joining (NHEJ) is the major mechanism for repairing DSBs (4). NHEJ involves at least six proteins: Ku70, Ku80, DNA-PKcs, DNA ligase IV, XRCC4, and Artemis. DNA ligase IV (LIG4) associates with XRCC4 during the final rejoining step of NHEJ (5).

LIG4 deficiency (OMIM 606593) is an extremely rare autosomal recessive disorder caused by mutations in the LIG4 gene. It is characterized by microcephaly, growth retardation, developmental delay, dysmorphic facial features, variable immunodeficiency, pancytopenia, a predisposition to malignancy, and pronounced clinical and cellular radiosensitivity (6).

The LIG4 gene maps to chromosome 13q33-q34; it contains two exons and comprises four domains: the DNA-binding domain (DBD), the nucleotidyltransferase domain (NTD), the oligo-binding domain (OBD), and the XRCC4-binding domain (XBD) (7). Our previous report identified p.R278L (c.833G>T) as a unique mutation site present in the majority of Chinese cases. We predicted that the p.R278L mutation is a hot spot or a founder effect in a Chinese population (8).

The founder effect is a genetic variation that occurs when a new population is created from a small number of individuals in a larger population (9). Ideally, the frequency of alleles in a population is distributed randomly between progeny; however, selection, variation, or inbreeding can affect the frequency of alleles in the progeny. A series of genetic markers that are inherited together through generations is called a haplotype; a haplotype demonstrates high linkage, resulting in little or no separation during meiotic recombination (10). Usually, the most common haplotype represents the polymorphism of most individuals within a population, which tends to be inherited as a whole by the offspring. Haplotype analysis can help confirm whether there is a founder effect in a mutation (11).

Here, we describe the clinical, immunological, and genetic characteristics of patients with LIG4 deficiency and investigate the phenotypic and mutation spectrum of the LIG4 deficiency to determine whether the p.R278L mutation is descended from a common ancestor *via* the founder effect.

## 2 Materials and Methods

### 2.1 Patients

Initially, all patients enrolled in the study were suspected of having combined immunodeficiency based on clinical manifestations, examination findings, and clinical laboratory results. Eight patients with LIG4 deficiency (P8 to P15) were recruited from the Children’s Hospital of Chongqing Medical University between 2016 and 2020. Seven patients (P1 to P7) that harbored the p.R278L mutation in the previous study were included (8); therefore, 15 patients were enrolled. The relevant clinical data are summarized in **Table 1A**. The assessment criteria for child growth and development published by WHO were used as a reference (12). Permission to participate in the study was provided by the patients’ families (all of whom provided informed consent), and the study was approved by the Medical Ethics Committee of Children’s Hospital of Chongqing Medical University.

**Table 1A:** Anthropometric data and clinical characteristics.

	Sex	Age of onset (months)	Age of diagnosis (months)	Family history	Gest/ weeks	BW/ s.d.(kg)	Anthropometric Data				Clinical Features							
							Age of examination (months)	OFC/s.d.(cm)	Height/s.d.(cm)	Weight/ s.d.(kg)	Form of onset	Development delay	Facial dysmorphia	Immunodeficiency	Etiology of infections	Malignancy	Others	Outcome
P1	F	11	18	_	38	−3.5(2.2)	18	−4.0 (40)	−3.0 (72)	−3.0(7.0)	Pneumonia, diarrhea	+	_	Chronic diarrhea, respiratory infection, thrush, sepsis, otitis media, onychomycosis	*Streptococcus pneumoniae*, CMV	Non-Hodgkin lymphoma	Pancytopenia	Die of malignancy At 3 years
P2	F	5	35	_	40	−1.5(2.9)	35	−4.0 (42)	−4.0 (80)	−4.5(7.5)	Pneumonia, diarrhea	+	_	Chronic diarrhea, respiratory infection, thrush, otitis media	*Streptococcus pneumoniae*, *Candida albicans*	_	Neutropenia and anemia, Colonoscopy: colitis	Die of severe Pneumonia at 3.5 years
P3	F	12	18	_	38	−3.0(2.4)	30	−5.0 (40)	−5.0 (72)	−5.0(7.0)	Pneumonia, diarrhea	+	_	Chronic diarrhea, pneumonia, otitis media, peritpnitis	*Streptococcus pneumoniae*, *Haemophilus influenza*	_	Anemia and thrombocytopenia, Inguinal hernia	Die of severe pneumonia at 3 years
P4	M	2	3	_	38	−2.5(2.5)	3	−3.0 (37)	−3.0 (55)	−3.5(3.0)	Pneumonia, diarrhea	+	_	Chronic diarrhea, severe pneumonia, ARDS	N/A	_	Anemia, atrial septal defect	Die of severe pneumonia at 4 months
P5	F	8	24	_	38	−4.0(2.0)	30	−4.5 (41)	−3.0 (80)	−5.5(6.0)	Diarrhea	+	+	Chronic diarrhea, severe Pneumonia	*Salmonella typhimurium*, *Pneumocystis carinii*	_	Pancytopenia, Phenylalanine dysmetabolism	Die of severe Pneumonia at 2.4 years
P6	F	10	18	+	39	−3.0(2.4)	21	−4.5 (40)	−5.0 (67)	−4.0(6.5)	Diarrhea	+	+	Chronic diarrhea, recurrent pneumonia	*Salmonella typhimurium*, *Enterobacter aerogenes*	_	AIHA and thrombocytopenia	Die of severe pneumonia at 2.5 years
P7	M	6	23	_	40	−2.5(2.6)	23	−7.0 (40)	−5.0 (70)	−5.5(5.0)	Pneumonia, Anemia	+	_	Chronic diarrhea, severe pneumonia, BCG infection, thrush	*Salmonella typhimurium*, CMV, acid-producing *Klebsiella*	–	Pancytopenia, Cytomegalovirus retinitis, and blindness	Die of severe pneumonia at 2 years
P8	M	8	18	_	38	−1.5(2.9)	18	−4.0 (42)	−4.0 (72)	−3.0(8.0)	Pneumonia, diarrhea	+	_	Chronic diarrhea, Recurrent pneumonia	*Salmonella typhimurium*, EBV	_	Pancytopenia	Die of severe pneumonia and diarrhea at 3 years
P9	F	2	12	+	36	−3.5(1.9)	13	−3.5 (40)	−3.0 (67)	−2.0(7.5)	Lymphocytopenia, thrombocytopenia	+	_	Chronic diarrhea, thrush and purpura	*Salmonella typhimurium*, *Candida albicans*	_	Pancytopenia	Die of HLH and sepsis at 3 years after HSCT
P10	M	1	12	_	38	−1.0(3.0)	11	−5.5 (39)	−2.0 (70)	−2.0(7.5)	BCG infection	_	_	Chronic diarrhea, respiratory infection and BCG infection	*Salmonella typhimurium*, BCG	_	Pancytopenia	HSCT at 2 years Survive
P11	M	4	28	_	38	−3.0(2.4)	35	−4.5 (43)	−3.0 (85)	−3.0(10.0)	Diarrhea	_	_	Chronic diarrhea	N/A	_	Pancytopenia	Survive
P12	M	23	36	_	38	−3.0(2.4)	36	−5.5 (42)	−3.0(84.5)	−4.0(8.5)	Neutropenia	_	+	Chronic diarrhea, severe pneumonia, wart	*Salmonella typhimurium*, EBV, CMV, *Candida albicans*	Large B-cell lymphoma	Neutropenia and anemia	Survive
P13	F	7	20	_	38	−2.0(2.5)	17	−4.0 (40)	−3.0 (72)	−5.0(5.0)	Diarrhea, thrush	+	+	Chronic diarrhea, severe pneumonia, thrush	*Enterobacter aerogenes*, *Salmonella typhimurium*, EBV, CMV, Parainfluenza virus	_	Pancytopenia	Survive
P14	M	22	48	_	40	−1.5(3.0)	48	−2.0 (47)	−1.0 (100)	1.0(18.5)	Pancytopenia	_	_	_	_	_	Pancytopenia	Survive
P15	M	6	12	+	38	-2.5(2.6)	12	−7.5 (36)	−3.0 (67)	−4.5(5.0)	Diarrhea	_	_	Chronic diarrhea, severe pneumonia, BCG infection	*Moraxella catalae*, BCG	_	AIHA, subglottic stenosis	Survive

Anthropometric data stated as Zscores (standard deviation from population mean for age and sex), actual measurements in brackets. F, female; M, male; Gest, gestation; BW, birth weight; OFC, occipitofrontal circumference; s.d., standard deviation; (−), negative; (+), positive; AIHA, autoimmune hemolytic anemia; ARDS, Acute Respiratory Distress Syndrome; BCG, Bacille Calmette-Guerin; CMV, cytomegalovirus; HSCT, hematopoietic stem cell transplantation; HLH, Hemophagocytic lymphohistiocytosis; N/A, not available.

### 2.2 Cell Preparation

Peripheral blood mononuclear cells (PBMCs) were isolated from freshly drawn heparin-treated blood by Ficoll density gradient centrifugation, as described previously (8).

### 2.3 Immunological Function Analyses

#### 2.3.1 Lymphocyte Subsets

Whole blood was used for standard flow cytometry multicolor analysis; staining of lymphocyte surface markers was performed after red cell lysis, as described previously (13). A total of 20 subpopulations were examined to analyze T and B lymphocyte subsets.

#### 2.3.2 T Cell Receptor Excision Circles and Kappa-Deleting Recombination Excision Circles

During T cell receptor rearrangement, excised DNA fragments create TRECs. During B cell maturation, KRECs are generated during kappa-deleting recombination allelic exclusion and isotypic exclusion of the light chain. TRECs reside within the chromosome, whereas KRECs are excised from genomic DNA. Quantification of TRECs and KRECs was performed using DNA samples extracted from peripheral blood. Quantification of TRECs and KRECs was performed by nested and quantitative real-time reverse transcription polymerase chain reaction (PCR) (qRT-PCR) (14, 15).

#### 2.3.3 CDR3 Spectratyping

Each T cell receptor (TCR) Vβ fragment was amplified using one of 23 Vβ-specific primers and a 5’FAM-labeled Cβ primer (16). The PCR products were sequenced by Sangon Biotech Company (Shanghai, China). The data were analyzed using Gene Mapper V3.5, and a scoring system was used to evaluate TCR Vβ diversity: a score <4 indicated a skewed subfamily (17, 18).

#### 2.3.4 Assessment of Maternofetal T Cell Engraftment

To detect the presence of maternofetal T cell transfusion, DNA samples obtained from each patient and their mother were subjected to short tandem repeat (STR) analysis by Kindstar Global Gene Technology Company (Wuhan, China).

#### 2.3.5 Proliferation of T Cell and B Cell

PBMCs were incubated with 1.25 μl/ml CFSE (Invitrogen) at 37°C. After 10 min, the cells were washed twice at 4°C with 5 ml Roswell Park Memorial Institute (RPMI) medium containing 10% fetal bovine serum (FBS). Cells were then resuspended in 600 μl of RPMI/10% FBS and seeded into 96-well plates along with 5 μg/ml phytohemagglutinin (PHA), 10 μg/ml lectin from pokeweed mitogen (PWM), and the same volume of RPMI for 72 h. After staining with CD3-PerCP (clone: HIT3a, BioLegend), CD4-PE-Cy7 (clone: RPA-T4, BioLegend), CD8-PE (clone: RPA-T8, BioLegend), and CD19-APC (clone: HIB19, BioLegend) antibodies, cells were analyzed and examined by flow cytometry (8).

### 2.4 LIG4 Mutation Analysis

Genomic DNA was extracted from peripheral blood leukocytes using a Gentra Puregene blood kit (Qiagen, Hilden, Germany). In some patients (P1–P4, P7, P10–P15), NGS of the family was performed firstly. The filtration of the WES data includes DNA library preparation, enrichment and sequencing of targeted genes, and bioinformatics analysis (MyGenostics, Beijing, China), as previously described (8). Genes associated with primary immunodeficiency diseases and other immune-related diseases had been updated according to the IUIS PID Classification Committee. In this study, four steps were used to select the potential pathogenic mutations in downstream analysis (i): Mutation reads should be more than 5, and mutation ration should be no less than 30% (ii); The mutations should be removed, when the frequency of mutation was more than 5% in 1,000 g, ESP6500, and Inhouse database (iii); The mutations should be dropped, if they were in InNormal database (MyGenostics) (iV); The synonymous mutations should be removed, when they were not in the HGMD database. After that, the rest mutations should be the potential pathogenic mutations for further analysis and judgment based on clinical phenotypes and phenotypic databases (OMIM and ClinVar). All mutations identified by NextSeq 500 sequencing were confirmed by Sanger sequencing. The coding exons and exon-intron boundaries of LIG4 were amplified by PCR. Both strands of the amplified PCR products were sequenced by Sangon Biotech Company. Whole-exon sequencing of P13 suggested that there might be copy number variation of exon. Therefore, the normal control samples, proband, and family samples were conducted by fluorescence quantitative PCR, and the copy number of the second exon of the target gene LIG4 was detected with the ALB gene as the internal reference gene. The pathogenicity of mutations was evaluated by four algorithms [PROVEAN (http://provean.jcvi.org/index.php), SIFT (http://sift.jcvi.org/), MutationTaster (http://www.mutationtaster.org/), and CADD (https://cadd.gs.washington.edu/snv)]. A scaled CADD PHRED of greater or equal 20 indicates the 1% most deleterious mutations in human genome. The frequency of mutations was searched in ChinaMap (http://www.mbiobank.com), gnomAD (https://gnomad.broadinstitute.org/), and DDBJ (https://www.ddbj.nig.ac.jp/index-e.html). The potential structural impact of the novel mutations was predicted by PymoL 2.1 program (https://pymol.org/2/).

### 2.5 The Expression of the Mutant Protein

Full-length wild-type (WT) LIG4 cDNAs was ordered from Youbio Biotech Company (Changsha, China). Mutant LIG4 cDNAs (p.R278L and p.R278H) were constructed by PCR mutagenesis and then subcloned into the p3xFlag-CMV-7.1 vector. In the overexpress system, HEK293T cells were transfected with 0.5 μg plasmids (WT, p.R278L, p.R278H), respectively. Cells were harvested 24 h after transfection, and then the expression of LIG4 was tested by Western blot with anti-LIG4 (EPR16531, Abcam) or anti-Flag antibody (2B3C4, proteintech).

### 2.6 Haplotype Analysis for LIG4 p.R278L Mutation

Haplotype analysis was performed to determine whether the LIG4 p.R278L mutation represents a founder mutation. Single-nucleotide polymorphisms (SNPs) were analyzed by Guoke Biotechnology Company (Beijing, China) using an Illumina Infinium^®^ Human Omni 2.5-8 v1.3 Bead-Chip (Illumina, San Diego, CA, USA). Eight SNPs [rs9301287, rs9559278, rs1931348, rs1931349, rs2391626, rs915047, KGP10207473 (rs9514825), and KGP9393242 (rs9520821)] within a genomic region of 500 kb around the LIG4 p.R278L mutation (rs104894421) and with linkage disequilibrium (LD) values >0.2 were selected based on the results of Plink linkage analysis. Variations between Chinese Han South (CHS) and Chinese Han Beijing (CHB) populations were downloaded from the 1000 Genomes Project (https://www.internationalgenome.org/) and used as “normal sample haplotype” information. The frequencies of the identified haplotypes were analyzed using the Haploview 4.2 program (https://www.broadinstitute.org/haploview/). A binomial probability formula was used to calculate the probability of a mutation occurring recurrently as a *de novo* event in the same haplotype.

### 2.7 Estimating the Age of the Mutation

To better understand its history, the age of the LIG4 p.R278L mutation was estimated using the DMLE 2.3 program (http://www.dmle.org/). This software uses the Markov chain Monte Carlo algorithm for Bayesian inference of mutation age based on the observed LD at multiple genetic markers. The population growth rate was set as 0.025, with an intergenerational time interval of 25 years. The disease sample ratio was 0.002, which is the software default parameter.

## 3 Results

### 3.1 Clinical Characteristics of Chinese Patients With LIG4 Deficiency

All 15 patients were from different families. Eight were male and seven were female. There was no evidence of potential skewing in the sex ratio. The clinical characteristics are listed in **Table 1A**. P6, P9, and P15 had a family history of early death or failed pregnancy (the older sister of P6 died of pneumonia at the age of 1 year, the older sister of P9 died of recurrent fever and diarrhea at the age of 8 months, and the mother of P15 had a previous pregnancy with embryo growth arrest). All were full-term infants, although five were small for their gestational age (SGA; i.e., birth weight 2 standard deviations below average).

The average age of symptom onset was 8 months (range, 1–23 months), and the median time of diagnosis was 18 months. The most common onset manifestations (soon after birth or later in life) included diarrhea, pneumonia, thrush, and BCG infection; two cases (P9 and P14) presented initially with hemolytic anemia.

Every patient except P14 suffered from chronic diarrhea, which aggravated their nutritional status and potentially contributed to growth failure. Pneumonia was the second most common and deadly type of infection. Salmonella was cultured from the stools of seven patients with diarrhea. The main etiological agent of respiratory tract infections was bacteria, including *Streptococcus pneumoniae*, *Enterobacter aerogenes, Moraxella catarrhiae, Acid-Producing Klebsiella*, and *Haemophilus influenzae*. The copy number of cytomegalovirus (CMV) in the blood of four patients was high (P1, P7, P12, and P13). BCG infection was suspected in P10 and P15; the BCG vaccination site was ulcerated, and adjacent lymph nodes were enlarged.

**Figure 1:**
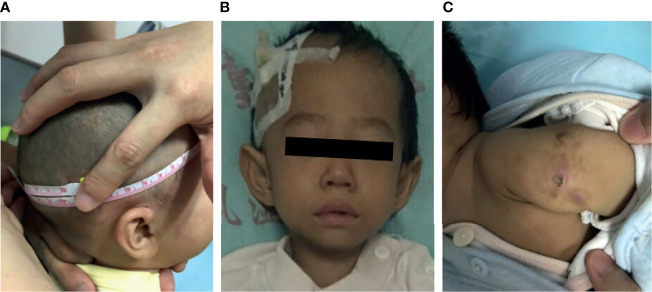
Clinical data for patients with LIG4 deficiency. **(A)** Microcephaly in patient P9. **(B)** Facial dysmorphism in patient P13. **(C)** BCG scar ulceration and exudation in patient P10.

Growth failure was evident in all patients. Occipitofrontal circumference, weight, and height were significantly below normal values (*p <*0.05). Data on head circumference at birth were scarce; however, our estimates were low because almost every patient presented at our hospital with a head circumference >3 SD below the population mean. A previous study in mice shows that LIG4 is essential for neuronal cell development. Consequently, most patients presented with short stature and microcephaly. Ten cases (P1–P9 and P13) showed developmental delay; all failed to achieve milestones of child development for their age group. P12 and P13 had a large nose, with a prominent nasal bridge as the facial dysmorphism (**Figure 1**).

All patients exhibited cytopenia (**Table 1B**). Leukocytes were the most affected cell type (11/15), followed by red blood cell. Three patients presented with pancytopenia. Bone marrow aspiration revealed active bone marrow hyperplasia with no morphological abnormalities, while showed failure bone marrow in biopsy. Notably, P14 manifested with cytopenia; initially, the bone marrow results led us to suspect that this patient had “myelodysplastic syndrome” (MDS) or “aplastic anemia” (AA). There was some evidence of autoimmunity. Coomb’s test was positive in four patients during the early course of the disease (P5, P6, P7, and P9). Two patients were positive for thyroid peroxidase antibodies (P9 and P10).

**Table 1B:** Hematological and immune investigations.

	Hematological test results					Lymphocyte subset cells/ul						Immunoglobulins (g/L)			TRECs (copies/reation)	KRECs (copies/reation)	Autoimmune antibodies
	WBC (10^9/L)	Neut	Lymph	Hb (g/L)	PLT (10^9/L)	CD3+	CD3+ CD4+	CD3+ CD8+	CD19+	CD16+ CD56+	γδT	IgG	IgA	IgM			
P1	2.55	68%	25%	90	30	164	14	88	2	69	N/A	1.92	0.01	1.45	N/A	N/A	N/A
P2	3.09	78%	16%	73	173	138	52	60	1	272	N/A	13.8 (IVIG)	0.067	0.22	N/A	N/A	N/A
P3	3.88	83%	11%	92	45	255	31	189	0	50	N/A	0.087	0.049	0.389	N/A	N/A	N/A
P4	5.05	35%	58%	78	283	1,020	216	464	31	1,916	N/A	0.921	<0.067	0.123	32	18	N/A
P5	2.89	56%	38%	97	7	513	22	394	0	16	N/A	9.17 (IVIG)	<0.067	0.663	10	7	Coomb’s test (+)
P6	3.59	68%	30%	84	67	161	37	88	1	540	N/A	10.2 (IVIG)	<0.067	0.217	106	2	Coomb’s test (+)
P7	3.25	27%	65%	95	80	1,739	316	1,265	285	1,165	N/A	<0.333	<0.067	<0.0417	N/A	N/A	Coomb’s test (+)
P8	6.21	58%	35%	71	5	1,694	320	1,155	8	1,450	962	1.12	<0.0667	1.54	2	5	N/A
P9	2.2	80%	6%	103	3	78	18	23	19	178	62	6.7 (IVIG)	0.37	1.03	0	0	Coomb’s test (+), TPOAb (+)
P10	2.15	74%	18%	95	276	170	23	63	1	94	112	8.24 (IVIG)	0.38	1.1	0	0	TPOAb (+)
P11	3.98	72%	20%	93	82	550	102	320	1	258	225	N/A	N/A	N/A	3	0	N/A
P12	1.69	69%	10%	90	200	151	72	43	12	190	55	6.6 (IVIG)	<0.001	<0.001	14	0	N/A
P13	1.42	14%	69%	70	40	1,012	177	556	1	495	N/A	1.46	0.51	1.54	0	0	N/A
P14	2.01	39%	29%	105	75	450	110	210	10	312	105	9.71	0.67	0.72	0	0	N/A
P15	7.4	51%	41%	105	370	1,288	230	510	3	1,737	1,081	5.4 (IVIG)	1.07	0.485	0	0	N/A

WBC, white blood count; Neut, neutrophil count; Lymph, Lymphocyte count; Hb, hemoglobin; PLT, platelet count; TRECs, T cell receptor excision circles; KRECs, kappa-deleting recombination excision; TPOAb, thyroid peroxidase antibodies; N/A, not available. Red means above the reference range, and blue means below the reference range.

P12 presented with hemocytopenia, characterized by chronic diarrhea and pneumonia. Abdominal imaging and biopsy (of the sigmoid colon) at the age of 4 years confirmed EBV-positive diffuse large B cell lymphoma (non-germinal center origin). Chemotherapy was not a treatment option due to pulmonary fungal infection and poor nutritional status.

Prior to diagnosis of LIG4 deficiency, P7 and P9 received steroids and rituximab to treat autoimmune hemolytic anemia (AIHA) and thrombocytopenia. P14 received cyclosporine for hemocytopenia; this patient was refractory to immunosuppressive therapy. Due to repeated infections, most patients received a variety of antibacterial or antiviral drugs, and some received antifungal or antituberculous drugs. However, P1–P8 died or stopped treatment without transplantation due to marked exacerbation of pulmonary infections and respiratory failure. Hematopoietic stem cell transplantation, the only effective radical cure for AIHA, was performed for P9 and P10. The donors were their respective parents (haploidentical donors) due to HLA matching difficulties; both engrafted successfully. After transplantation, mycophenolate mofetil, tacrolimus, MTX, and steroids were used for GVHD prophylaxis. Unfortunately, P9 developed recurrent fever 3 months after transplantation, thought to be due to EBV-driven post-transplantation lymphoproliferative disease and hemophagocytic lymphohistiocytosis (HLH). She received the HLH-2008 chemotherapy regimen (including two doses of VP16) but died of sepsis 3 months later. Notably, HCT did not cure microcephaly and neurodevelopmental delay in P10. The remaining patients (P11–P15) have not received HCT, but continue to survive; all receive IVIG and oral co-trimoxazole to prevent infection (standard treatments after a diagnosis of LIG4 deficiency).

### 3.2 Immune Characteristics

Immunological function analyses were also performed (**Table 1B**). Flow cytometry analysis of peripheral blood showed a significant reduction in the absolute numbers of CD19+ B cell and CD3+ T cell; however, NK cell (T-B-NK+ phenotype) counts were near-normal. Seven of them (P8, P9, P10, P11, P12, P14, and P15) were analyzed in detail. Two patients (P8 and P15) exhibited a marked increase in the number of γδ T cell compared with healthy children. IgG levels in six of the 14 patients (except the P11) were significantly lower than the normal reference value (*p <*0.05). Others had normal levels of IgG; however, they were tested after receiving intravenous immunoglobulin. The TRECs and KRECs count was significantly below the detection limit, although the lymphocyte counts in P8 and P15 were not that low (19). STR analysis of eight patients ruled out maternofetal transfusion (P8–P15).

**Figure 2:**
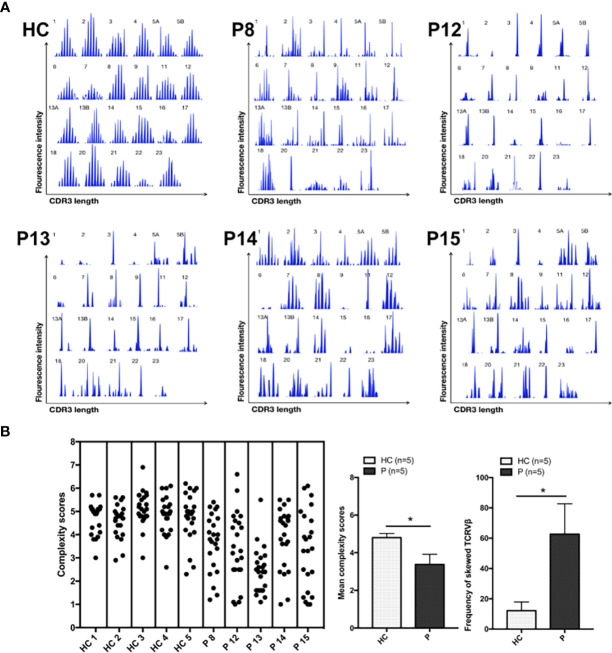
TCR-Vβ analysis. **(A)** All 23 TCR-Vβ subfamilies in the healthy controls exhibited a Gaussian distribution. CDR3 size distribution of the TCR-Vβ subfamilies in patients P8, P12, P13, P14, and P15. **(B)** The complexity scores for each healthy control subject (left) and patient (right) are shown. The mean (range) complexity scores of the patient and control groups are shown in the histogram. The frequencies of TCR-Vβ subfamilies with skewed CDR3 length patterns are shown in the histogram, as determined by a complexity score <4. HC, healthy control subjects; P, patients. *p < 0.05.

Analysis of TCR-Vβ diversity was performed in five newly enrolled patients (P8, P12, P13, P14, and P15). Most TCR-Vβ subfamilies exhibited monoclonal or oligoclonal peaks, and TCR repertoire complexity was limited, a finding similar to that in our previous report (8). P14 and P15 exhibited less skewed TCR diversity than healthy controls, suggestive of less severe impairment of V(D)J recombination and TCR function. In those age-matched healthy controls, the majority of the 23 TCR-Vβ subfamilies exhibited a Gaussian curve with 6–9 peaks, reflecting a polyclonal Vβ repertoire. The frequency of skewed TCR-Vβ subfamilies in the patients was higher than that in the healthy control (**Figure 2**).

When PBMCs from patients were stimulated with PHA for 3 days, the percentages of proliferating CD4+ and CD8+ T lymphocytes were 0.2 and 1.9% in P8; 2.78 and 4.33% in P11; 3.79 and 1.67% in P12; and 1.1 and 8.3% in P14, respectively. CFSE fluorescence histograms generated by flow cytometry revealed no obvious peak with respect to cell division. The percentages of proliferating CD4+ and CD8+ T lymphocytes in the PBMCs population from the normal control were 71.9 and 65.6%, respectively, with obvious peaks in cell division. Taken together, these data suggest that T cell proliferation in the LIG4 deficiency patients was severely impaired. B cell proliferation was analyzed only in P11. Proliferation of CD19+ B cell after stimulation by PWM was 4.93% in the patient and 47.8% in the normal control.

### 3.3 Genetic Characteristics

Patients with LIG4 deficiency came from 11 different provinces in China. Mutations in the LIG4 gene were detected by either Sanger or next-generation sequencing. According to the clinical manifestations of microcephaly, immune deficiency, and autosomal recessive inheritance pattern, LIG4 was finally identified as the sole pathogenic gene. No other PID genes in the 2019 update of the IUIS IEI classification were found in each patient. Fourteen different mutations were identified, three of which have not been described previously. Most were compound heterozygous mutations, while P3 and P8 harbored a homozygous p.R278L. Most mutations were predicted to be “Deleterious,” “Damaging,” “Disease causing,” or “Prediction disease causing” by different algorithms. Frameshift mutations and copy number variants cannot evaluated by CADD. All CADD PHRED score of the missense or non-sense mutations were >20 and regarded as deleterious except p.T9I. The allele frequency of p.T9I was about 20% in ChinaMap and was predicated as SNP by Mutation Taster. Mutation p.R278L was found in ChinaMap (6/21176) and gnomAD (1/1558, East Asian) but not in DDBJ or other population (**Table 2**).

**Table 2:** Genetic characteristics.

Nucleotide change		Protein change	Father	Mother	Province of Origin	Prediction				Frequency (ChinaMap)	
						PROVEAN	SIFT	Mutation Taster	CADD PHRED	Allele Frequency	Count
P1	c.833G>T	p.R278L	p.R278L		Zhejiang	Deleterious	Damaging	Disease causing	26.0	0.00028334	6/21176
	c.1271-1275delAAAGA	p.K424RfsX20		p.K424RfsX20		N/A	N/A	Prediction disease causing	N/A	0.000472233	10/21176
P2	c.833G>T	p.R278L		p.R278L	Sichuan	Deleterious	Damaging	Disease causing	26.0	0.00028334	6/21176
	c.1271-1275delAAAGA	p.K424RfsX20	p.K424RfsX20			N/A	N/A	Prediction disease causing	N/A	0.000472233	10/21176
P3	c.833G>T	p.R278L	p.R278L		Guizhou	Deleterious	Damaging	Disease causing	26.0	0.00028334	6/21176
	c.833G>T	p.R278L		p.R278L		Deleterious	Damaging	Disease causing	26.0	0.00028334	6/21176
P4	c.833G>T	p.R278L	N/A	N/A	Shanxi	Deleterious	Damaging	Disease causing	26.0	0.00028334	6/21176
	c.2113G>T	p.E705X				N/A	N/A	Disease causing	34.0	N/A	N/A
P5	c.833G>T	p.R278L	p.R278L		Tianjin	Deleterious	Damaging	Disease causing	26.0	0.00028334	6/21176
	c.26C>T	p.T9I		p.T9I		Deleterious	Damaging	Ploymorphism	18.9	0.2066696	4377/21176
	c.1142-1143delCT	p.L382EfsX4		p.L382EfsX4		N/A	N/A	Prediction disease causing	N/A	0.000236116	5/21176
P6	c.833G>T	p.R278L	p.R278L		Beijing	Deleterious	Damaging	Disease causing	26.0	0.00028334	6/21176
	c.935delC	p.P313HfsX19		p.P313HfsX19		N/A	N/A	Prediction disease causing	N/A	N/A	N/A
P7	c.833G>T	p.R278L	p.R278L		Inner Mongolia	Deleterious	Damaging	Disease causing	26.0	0.00028334	6/21176
	c.2134-2135delTA	p.I712AfsX5		p.I712AfsX5		N/A	N/A	Prediction disease causing	N/A	N/A	N/A
P8	c.833G>T	p.R278L	p.R278L		Sichuan	Deleterious	Damaging	Disease causing	26.0	0.00028334	6/21176
	c.833G>T	p.R278L		p.R278L		Deleterious	Damaging	Disease causing	26.0	0.00028334	6/21176
P9	c.833G>T	p.R278L		p.R278L	Jiangxi	Deleterious	Damaging	Disease causing	26.0	0.00028334	6/21176
	c.1271-1275delAAAGA	p.K424RfsX20	p.K424RfsX20			N/A	N/A	Prediction disease causing	N/A	0.000472233	10/21176
P10	c.1296A>T	p.K432N	p.K432N		Henan	Deleterious	Damaging	Disease causing	23.7	N/A	N/A
	c.1672C>T	p.Q558X		p.Q558X		N/A	N/A	Prediction disease causing	40.0	N/A	N/A
P11	c.833G>T	p.R278L		p.R278L	Henan	Deleterious	Damaging	Disease causing	26.0	0.00028334	6/21176
	c.34T>A	p.S12T	p.S12T			Neutral	Damaging	Disease causing	21.1	0.00056679	12/21176
	c.2710C>T	p.Q904X	p.Q904X			N/A	N/A	Disease causing	38.0	N/A	N/A
P12	c.833G>T	p.R278L		p.R278L	Hubei	Deleterious	Damaging	Disease causing	26.0	0.00028334	6/21176
	c.1271-1275delAAAGA	p.K424RfsX20	p.K424RfsX20			N/A	N/A	Prediction disease causing	N/A	0.000472233	10/21176
P13	c.833G>T	p.R278L		p.R278L	Guizhou	Deleterious	Damaging	Disease causing	26.0	0.00028334	6/21176
	loss exon2	loss exon2				N/A	N/A	N/A	N/A	N/A	N/A
P14	c.980T>G	p.I327S		p.I327S	Henan	Deleterious	Tolerated	Disease causing	26.7	N/A	N/A
	c.2585_2586del	p.H862RfsX6	p.H862RfsX6			N/A	N/A	Prediction disease causing	N/A	N/A	N/A
P15	c.833G>T	p.R278L	p.R278L		Hunan	Deleterious	Damaging	Disease causing	26.0	0.00028334	6/21176
	c.1271-1275delAAAGA	p.K424RfsX20		p.K424RfsX20		N/A	N/A	Prediction disease causing	N/A	0.000472233	10/21176

N/A, not available.

**Figure 3:**
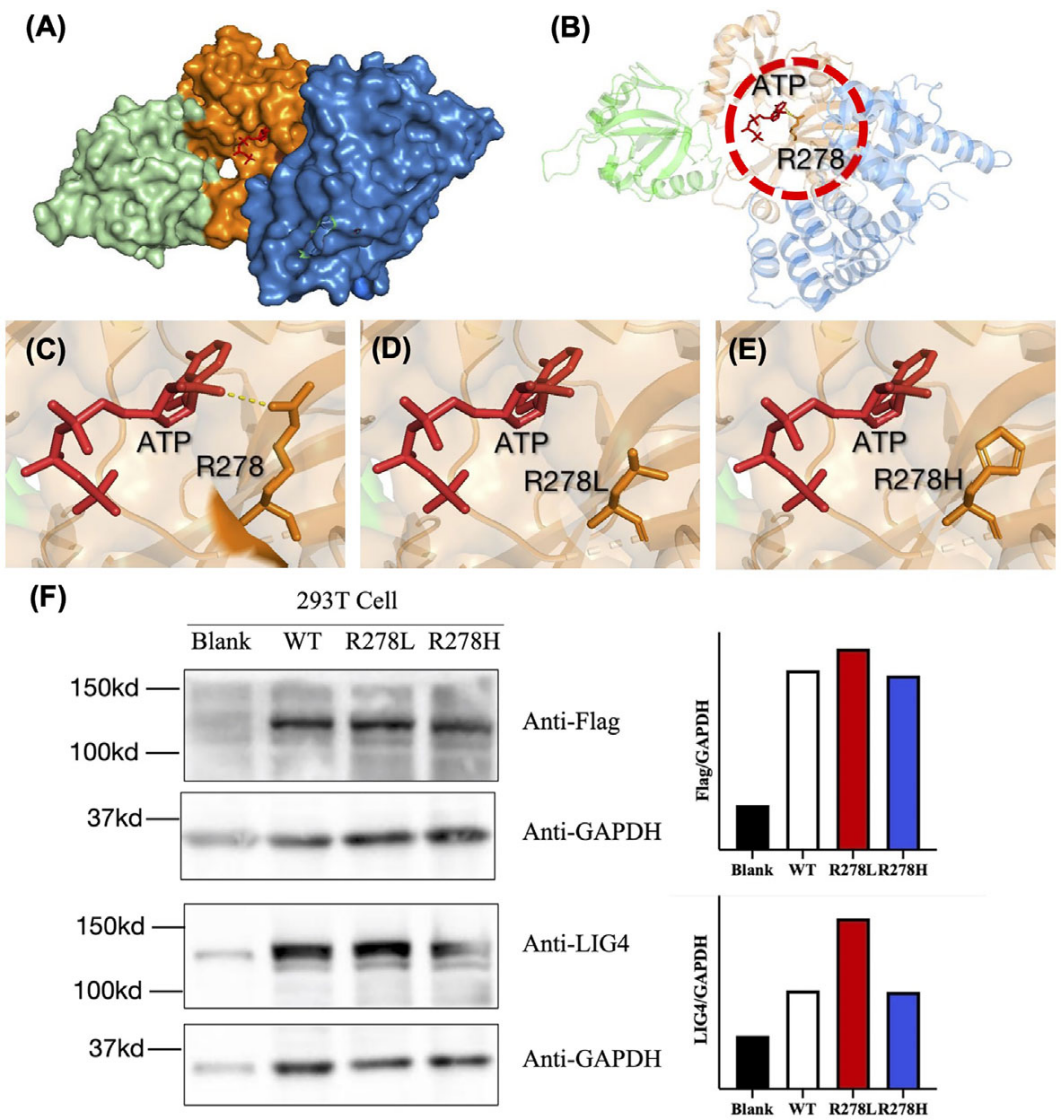
**(A)** Structural characterization of the catalytic domain of human DNA LigIV (PDB ID code 3W5O). The domains are shown in blue (DBD), orange (NTD), and cyan (OBD). The structure surrounding by Arg-278 (indicated by the dashed circle in **(B)** is enlarged. **(C–E)** The interaction between ATP and amino acids R278, R278L, and R278H was drawn using a tool packaged in PymoL. **(F)** Western blot analysis of the LIG4 protein expression in the HEK293T cells transfected with WT or mutant LIG4 plasmid (p.R278L or p.R278H).

**Figure 4:**
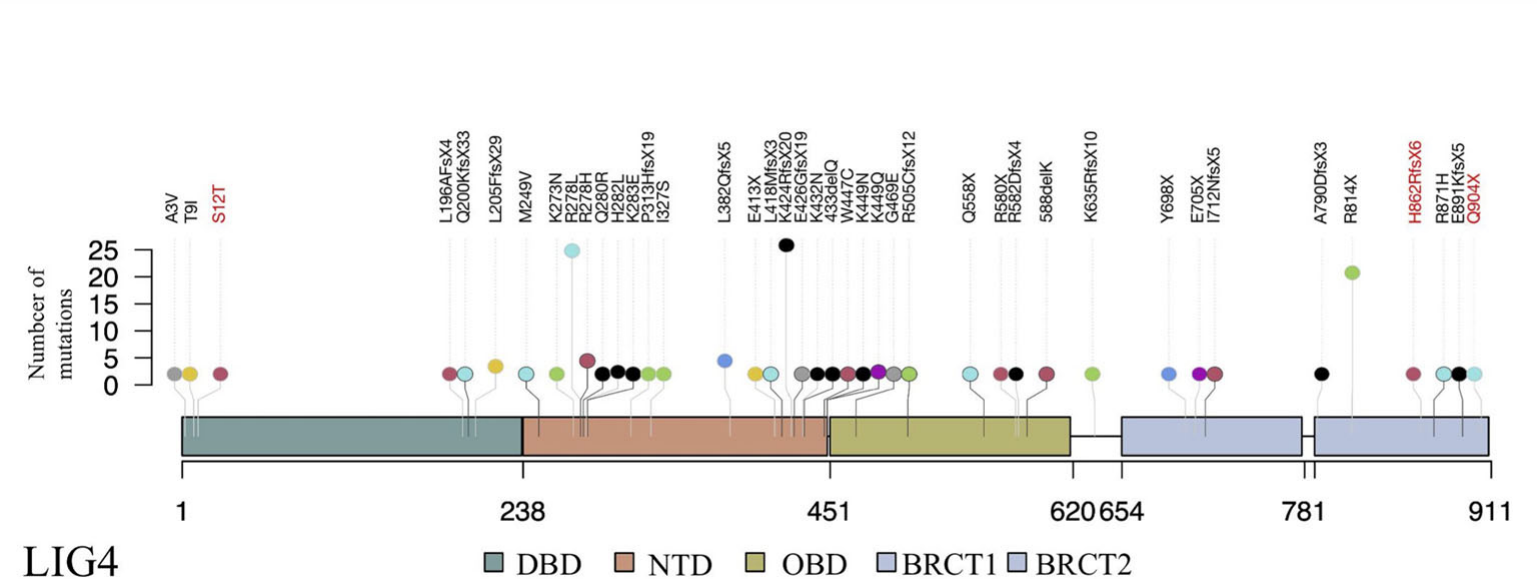
Schematic presentation of the LIG4 gene structure and a summary of mutation sites. The novel mutation sites identified in this study are shown in red. The height of the line represents the number of mutations. DBD, DNA-binding domain; NTD, nucleotidyltransferase domain; OBD, oligo-binding domain; XBD, XRCC4-binding domain.

Since recurrent infection is the predominant feature in these patients, and antibody deficiency and lymphocytopenia are present as a combined immunodeficient immunophenotype, we hypothesized that these mutations are loss of function. Since it was difficult to get enough primary cell from patients, we tried to build the KO fibroblast cell line several months ago. However, the cell line did not proliferate after LIG4 gene knockout, and we failed to get the KO clone. We transfected the mutant LIG4 (p.R278L and p.R278H) to HEK293 as an overexpression system and found that the mutant protein expressed comparably to wild type. To visualize the function of R278 at the amino acid level, we constructed a diagram to describe the structure of the DBD domain of LIG4, in which the main role of R278 is to bind ATP (**Figure 3**). The map reveals that mutations in R278 affect the activity of enzymes by altering the spatial conformation and the ability to bind ATP. LIG4 deficiency patients carrying the p.R278L mutation were concentrated in the Yangtze River valley of China. All mutation sites are shown in the simulation diagram (**Figure 4**) (20–37). Mutation p.R278L is a hot spot found only in Chinese patients. It is worth mentioning that the p.R278L mutation was also detected in P13; however, the ratio of the copy number of exon 2 of the LIG4 gene to that of the normal control was about 0.5 (**Supplementary Figure**), suggesting large deletion of heterozygosity in exon 2 of the LIG4 gene on the allelic chromosome. Another common genotype is p.K424RfsX20, which occurs in non-Chinese cases.

### 3.4 Shared Haplotype and Estimated Age of the Mutation

**Figure 5:**
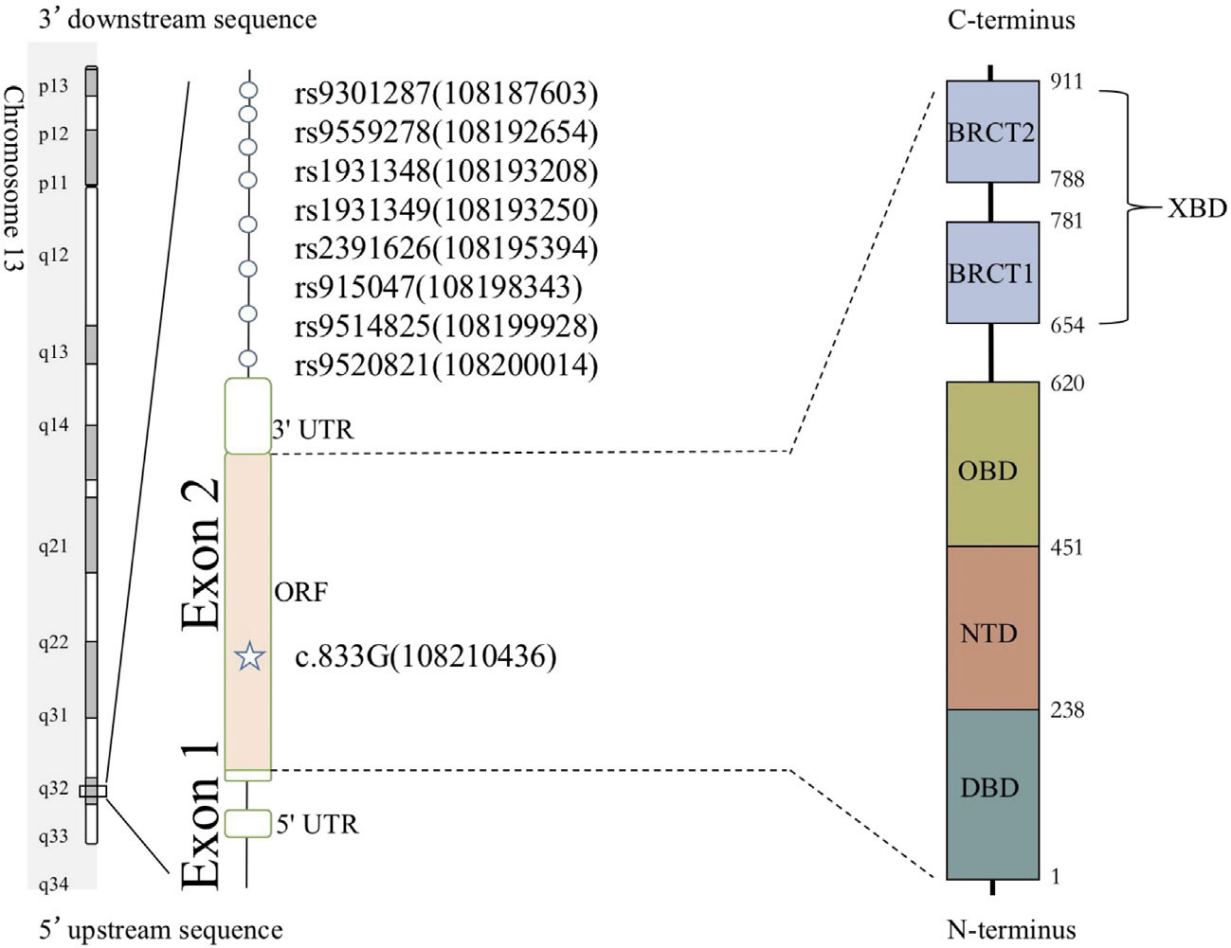
Eight SNPs were used to reconstruct the haplotypes for the LIG4 mutation c.833G. The positions of genetic markers (shown in brackets) were defined according to GRCh38.

**Figure 6:**
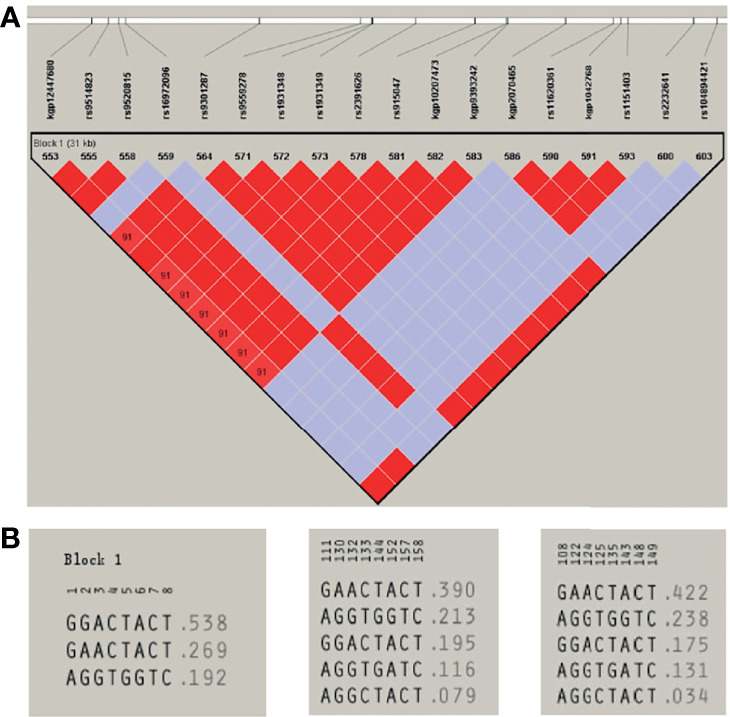
**(A)** Haploview-generated linkage disequilibrium (LD) patterns for the LIG4 mutation c.833G>T (p.R278L) and the predicted block structure. The rate of LD is represented by different colors (the highest rate of LD is red, the lowest in purple). **(B)** Potential haplotypes of the eight selected SNPs genotyped in our study in Patients (left) and in CHS (middle) and CHB (right) populations.

A total of eight unrelated LIG4 deficiency patients harboring the p.R278L mutation were included for haplotype analysis (P1, P4, P5, P6, P8, P9, P11, and P12). Haplotypes were characterized using a set of eight SNPs flanking the c.833G>T (p.R278L) mutation, with a length of approximately 2.3 kb (**Supplementary Table** and **Figure 5**). Using the Haploview program, a block of LD was located beside the LIG4 gene; three haplotypes were identified in the patients and five in the healthy control population. Haplotype analysis shows that haplotype GGACTACT was the most common in the patient group (53.8%), but occurred in only 19.5% of the CHS and 17.5% of the CHB population (**Figure 6**). The haplotype of the p.R278L mutation site is significantly different from that of the normal allele.

**Figure 7:**
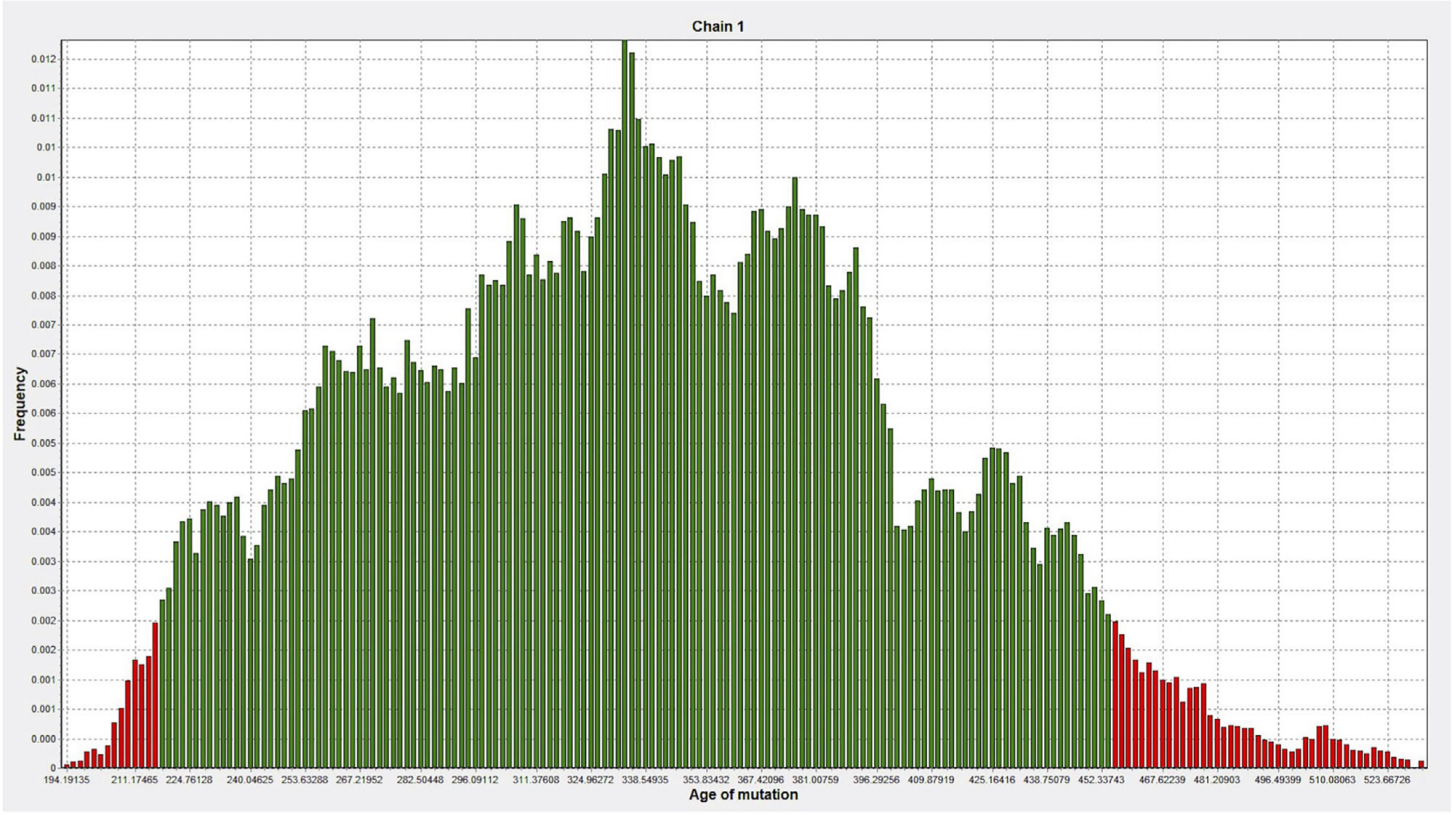
The age of the mutation was simulated 100,000 times by DMLE+2.3. The abscissa represents the age of mutation, in units of generation (about 25 years), and the ordinate represents the frequency (Green, within the 95% confidence interval of posterior distribution; Red, outside the 95% confidence interval).

DMLE+ 2.3 was used to analyze the age of the mutation; DMLE+ 2.3 provides the posterior distribution probability of the age of the mutated haplotype (38). Based on an intergeneration time of 25 years, it predicted that the age of the mutation is 353 generations (95% credible set; 217–454 generations), i.e., 8,825 years (95% credible set: 5,425–11,350 years) (**Figure 7**).

## 4 Discussion

According to the IEI classification standard updated by IUIS in 2019, LIG4 deficiency is a type of severe combined immunodeficiency (SCID) defined by CD3/CD19 lymphopenia, which affects cellular and humoral immunity (39). As such, LIG4 deficiency has a broad-spectrum phenotype that includes immunodeficiency, microcephaly, growth failure, facial dysmorphism, malignancy predisposition, and cellular sensitivity to ionizing radiation. Additional features include bony deformations such as syndactyly and congenital hip dysplasia (6).

Due to combined immunodeficiency, approximately three quarters of LIG4 deficiency patients suffer from recurrent infections, with varying degrees of severity. Among them, sinopulmonary infections are the most common (20). By contrast, the most common manifestation in our patient cohort was severe gastrointestinal tract infection. Chronic diarrhea and recurrent pneumonia were common at onset in almost all patients, leading to multiple hospital admissions and failure to thrive. Causative pathogens included bacteria, viruses, and fungi. Intestinal infection by *Salmonella typhimurium* is a unique manifestation in Chinese patients, indicating that gastrointestinal prophylaxis is of great importance. BCG is a unique pathogen associated with live attenuated vaccinations. Thus, a delayed BCG vaccination, or an IPV vaccination plan within 1 month after birth, should be considered as a replacement for the live attenuated vaccination plan.

It is classified as a severe immunodeficiency, and our patients mainly present severe immunodeficiency for loss of LIG4 function, whereas P14 had no history of recurrent or severe infection; the only manifestation was cytopenia. Initially, we suspected “MDS” or “AA” and so treated the patient with cyclosporine for 2 years, even though two compound heterozygous mutation sites in LIG4 gene had been detected before he came to our hospital. The p.I327S mutation in P14 was also detected in another LIG4 deficiency patient with pancytopenia and lymphoma, whereas the other mutation (p.H862RfsX6) has not been reported previously. After hospitalization at our center, laboratory tests confirmed the pathogenicity of these mutations by indicating a significant decrease in TRECs/KRECs and T lymphocyte proliferation, the TCR diversity is not so severely impaired as the other patients. The p.H862RfsX6 mutation located in the XBD causes a premature stop codon six amino acids downstream of the C-terminus of the LIG4 protein. Previous studies show a genotype-phenotype correlation with respect to the position of truncating mutations corresponding to disease severity. Transcripts encoding truncated proteins are predicted to be expressed at normal levels (21). We speculate that P14 present as hypomorphic phenotype since the mild clinical manifestation, less skewed TCR diversity, higher lymphocyte and immunoglobulin due to heterogenous p.I327S missense mutation.

Extreme growth failure and microcephaly is a common and early presentation of LIG4 deficiency patients. Murray et al. even screened some LIG4 deficiency patients for microcephalic primordial dwarfism before making a diagnosis of CID or SCID (21). This may be due to accumulation of DNA-DSB over time resulting in reduced lymphocyte production. Most of our patients were full-term infants, although they were SGA, which suggests intrauterine growth retardation. A previous study in mice shows that LIG4 is essential for neuronal cell development (40). LIG4 deficiency may lead to impaired prenatal differentiation of neuronal cell and result in microcephaly and developmental delay. Thus, significant microcephaly and growth restriction are regarded as the most prominent features of LIG4 deficiency patients. Developmental delay also occurred in five of our patients. “Bird-like” or “Seckel syndrome-like” traits are always observed in LIG4 deficiency patients (20). In this study, P12 and P13 had a large nose with a prominent nasal bridge; however, there were no other facial dysmorphisms such as a low anterior hairline, bilateral epicanthic folds, or up slanting palpebral fissures (6).

The symptomatic treatment of LIG4 deficiency patients includes long-term antibiotics, antiviral and antifungal chemoprophylaxis, immunoglobulin infusion, transfusion support, and avoidance of unnecessary exposure to ionizing radiation. HCT is a curative treatment for CID and SCID immunophenotypes and might reduce the risk of developing lymphoid malignancy. Of note, reduced-intensity conditioning regimens with low-dose Cyclosporin A should be considered due to radiosensitivity (6). However, HCT does not cure microcephaly or neurodevelopmental delay. Short stature and mild to moderate intellectual disability may remain. Extra social care is required to maintain a good quality of life, including attendance at a special school for intellectual disability (21). In our study, nine of the 15 patients died. In another cohort of LIG4 deficiency cases in China, four of seven died and two were lost to follow-up (37). There were another four patients in the other papers who were also lost to follow-up, and the possibility of death was high (35, 36). Thus in China, improvement of the early diagnosis and adequate treatment are necessary to improve the poor prognosis of LIG4 deficiency.

Due to disruption of V(D)J recombination, rearrangement of T and B lymphocyte receptors is aberrant, resulting in combined immunodeficiency and a skewed TCR-Vβ repertoire, as confirmed in the new cohort patients (P8–P15). Therefore, most LIG4 deficiency patients present with T-B-NK+ phenotype SCID, along with reduced antibody production. Flow cytometry analysis showed that reduced B cell counts are more pronounced than reduced T cell counts due to B cell development is more reliant on V(D)J recombination, maternofetal transfusion, or a spontaneous somatic reversion (8). However, STR confirmed no maternal engraftment in our eight patients (P8–P15), so maternofetal transfusion is not as common as in X-SCID patients. Also, there was no reduction in γδT cell. Previous studies argue that limited V(D)J recombination activity may provide γδ T cell with a developmental advantage (22, 41). LIG4 has also been shown to be involved in immunoglobulin class switch recombination (6). Thirteen of 14 (93%) and nine of 14 (64%) patients had low IgG and IgA levels, respectively, whereas eight of 14 patients (57%) had normal IgM levels.

Genetic analysis revealed that most Chinese LIG4 deficiency cases harbor the p.R278L mutation, which is unique and represents a mutational hot spot in China. There is clinical evidence for the pathogenicity of this mutant site. Another mutation in the same codon, which causes a different amino acid substitution (p.R278H) (40, 42), was reported only in a non-Chinese ethnic population. According to the pathogenicity prediction software, both of these mutations cause functional loss by affecting binding to ATP, although this needs to be confirmed. The P3 and P8 with homogeneous R278L manifested as severe infection, very low T/B cell counts, low TRECs, as well as normal protein expression (**Figure 3F**), indication p.R278L as loss of function mutation.

The high frequency and the geographic clustering of the LIG4 p.R278L mutation may be a hot spot or even a founder mutation. According to the principle of LD, haplotypes reflect the genetic information at the mutation site since they tend to be passed on to offspring as a whole. For example, haplotype analysis revealed three founder mutations (BRCA1 c.68_69delAG, c.5266dupC, and BRCA2 c.5946delT) in the Ashkenazi Jewish population, and a recurrent F8 mutation (c.6046C>T) causing hemophilia A in a northern Italian population (43, 44). In this study, we constructed five different haplotypes to explore the origin of the alleles carrying the p.R278L mutation in our LIG4 deficiency patients. Haplotype analysis identified only three haplotypes in LIG4 deficiency patients, the most common being haplotype GGACTACT (53.8%); this haplotype was much less common in the controls (CHS, 19.5%; CHB, 17.5%). The different frequencies of haplotypes, along with reduced genetic diversity, are typical features of isolated and stable populations. Thus, it is plausible that the LIG4 p.R278L mutation frequency increased in China due to a local founder effect.

There has been much debate about the origin of mankind. In recent years, a large amount of genomic data has been obtained and accumulated. The completion of the human genome project in 2003, the launch of the 1000 Genomes Project in 2008, and the study of ancient DNA have provided new clues that explain the evolution of the genetic structure of populations (45, 46). The recent African origin model hypothesis states that *Homo erectus*, the common ancestor of early *Homo sapiens* or Archaic humans, originated in Africa and then spread from the continent to other parts of the world about 2 million years ago. India is one of the many crossroads in the history of mankind (47, 48). Interestingly, when we analyzed this haplotype (GGACTACT) among other ethnic groups from the 1000 Genomes Project, we found that Bangladeshi and Pakistani (PJL), as well as the Indian populations in the United States (GIH) and England (ITU), also had the highest frequency of this haplotype. Therefore, we cannot exclude that this mutation might have spread from South Asia *via* migration (49–51). However, no LIG4 deficiency patients of Bangladeshi, Pakistani, or Indian origin have been reported.

Regarding the age of the common ancestor, the DMLE estimates that the LIG4 p.R278L mutation is approximately 8,825 years old. This corresponds to the Neolithic age, the period during which agriculture began in settled communities. The distribution patterns of such founder mutations might help determine the migration pathways of populations with Asian ancestry (48, 49). However, estimating the age of founder mutations will always be an inexact endeavor. The true recombination and mutation history of the relevant chromosomal segments is unknown. Thus, further studies on the prevalence of shared mutations in Asian countries, and haplotype analyses in other ethnic groups, would be helpful if we are to trace the common ancestor.

This study has some limitations. We did not confirm the DNA-repair defect in primary cell due to lack of PBMCs from patients. We also did not confirm it by other strategy since the fibroblast cell line did not proliferate after LIG4 gene knockout as well as the technical difficulties of recombinant LIG4 enzyme activity analysis. It could be overcome by primary cell from a newly diagnosed patient in the future.

## 5 Conclusion

In summary, LIG4 deficiency is a rare disease with a broad spectrum of presentations. The severity fluctuates greatly. In China, improvement of the early diagnosis and adequate treatment are necessary to improve the poor prognosis of LIG4 deficiency, and HCT is urgent. Pedigree analysis and haplotype construction revealed conservation of a single haplotype surrounding the p.R278L mutation, suggesting that this allele has a common ancestor. The finding of a founder effect in a highly recurrent mutation in a rare disease suggests that implementation of protocols for genetic diagnosis and for genetic counseling of affected pedigrees is essential. Also, the search for new targeted therapies such as base editing should be prioritized.

## Data Availability Statement

The datasets presented in this study can be found in Genebank with the following accession numbers:

BankIt2490138 ZJ-R278L MZ766072
BankIt2490148 QWY-R278L MZ766073
BankIt2490148 QWY-E705X MZ766074
BankIt2490153 GYT-R278L MZ766075
BankIt2490153 GYT-I712Afs MZ766076
BankIt2490153 XJ-R278L MZ766077
BankIt2490153 TKY-R278L MZ766078
BankIt2490153 TKY-K424Rfs MZ766079
BankIt2490153 DCZ-R278L MZ766080
BankIt2490153 DCZ-K424Rfs MZ766081
BankIt2490153 DYC-I327S MZ766082
BankIt2490153 DYC-H862Rfs MZ766083
BankIt2489891 TSY MZ825311
BankIt2490111 TSY-K424Rfs MZ825312
BankIt2490126 SXY-R278L MZ825313
BankIt2490126 SXY-K424Rfs MZ825314
BankIt2491465 YR-T9I MZ825315
BankIt2491472 YR-R278L MZ825316
BankIt2491472 YR-L382Efs MZ825317
BankIt2491472 WYH-R278L+P313HfsMZ825318
BankIt2491472 ZHR-K432N MZ825319
BankIt2491472 ZHR-Q558X MZ825320
BankIt2491472 DCY-R278L MZ825321
BankIt2491472 LC-R278L MZ825322
BankIt2491472 LC-K424Rfs MZ825323
BankIt2491472 ZKY-R278L MZ825324
BankIt2491472 ZKY-P904X MZ825325
BankIt2491472 ZKY-S12T MZ825326

## Ethics Statement

The studies involving human participants were reviewed and approved by the Medical Ethics Committee Children’s Hospital of Chongqing Medical University. Written informed consent to participate in this study was provided by the participants’ legal guardian/next of kin. Written informed consent was obtained from the individual(s), and minor(s)’ legal guardian/next of kin, for the publication of any potentially identifiable images or data included in this article.

## Author Contributions

XL, YA, and XZ designed experiments and analyzed the data. XL wrote the first draft of the manuscript and performed the experiments. YD provided control specimens from normal healthy children. QL, JJ, WT, LZ, JY, and XT contributed to scientific discussion, data interpretation, and revision of the manuscript. XZ designed the research, supervised the study, and revised the manuscript. All authors contributed to the article and approved the submitted version.

## Funding

This work was supported by the Natural Science Foundation of China (81471619, 82070135) and by Chongqing Technology Innovation and Application Demonstration (cstc2018jscx-msybX0005).

## Acknowledgments

We thank our colleagues at the Chongqing Key Laboratory of Child Infection and Immunity. We are grateful to our patients and their families for their cooperation.

## Conflict of Interest

The authors declare that the research was conducted in the absence of any commercial or financial relationships that could be construed as a potential conflict of interest.

## Publisher’s Note

All claims expressed in this article are solely those of the authors and do not necessarily represent those of their affiliated organizations, or those of the publisher, the editors and the reviewers. Any product that may be evaluated in this article, or claim that may be made by its manufacturer, is not guaranteed or endorsed by the publisher.
