# Supplementary material for: Characterization of a Cohort of Patients With LIG4 Deficiency Reveals the Founder Effect of p.R278L, Unique to the Chinese Population
Source: Front Immunol. 2021 Sep 24;12:695993. doi: 10.3389/fimmu.2021.695993 (PMC8498043; doi:10.3389/fimmu.2021.695993)
Supplement: Supplementary file 1 [file DataSheet_1.doc]

Supplementary Material


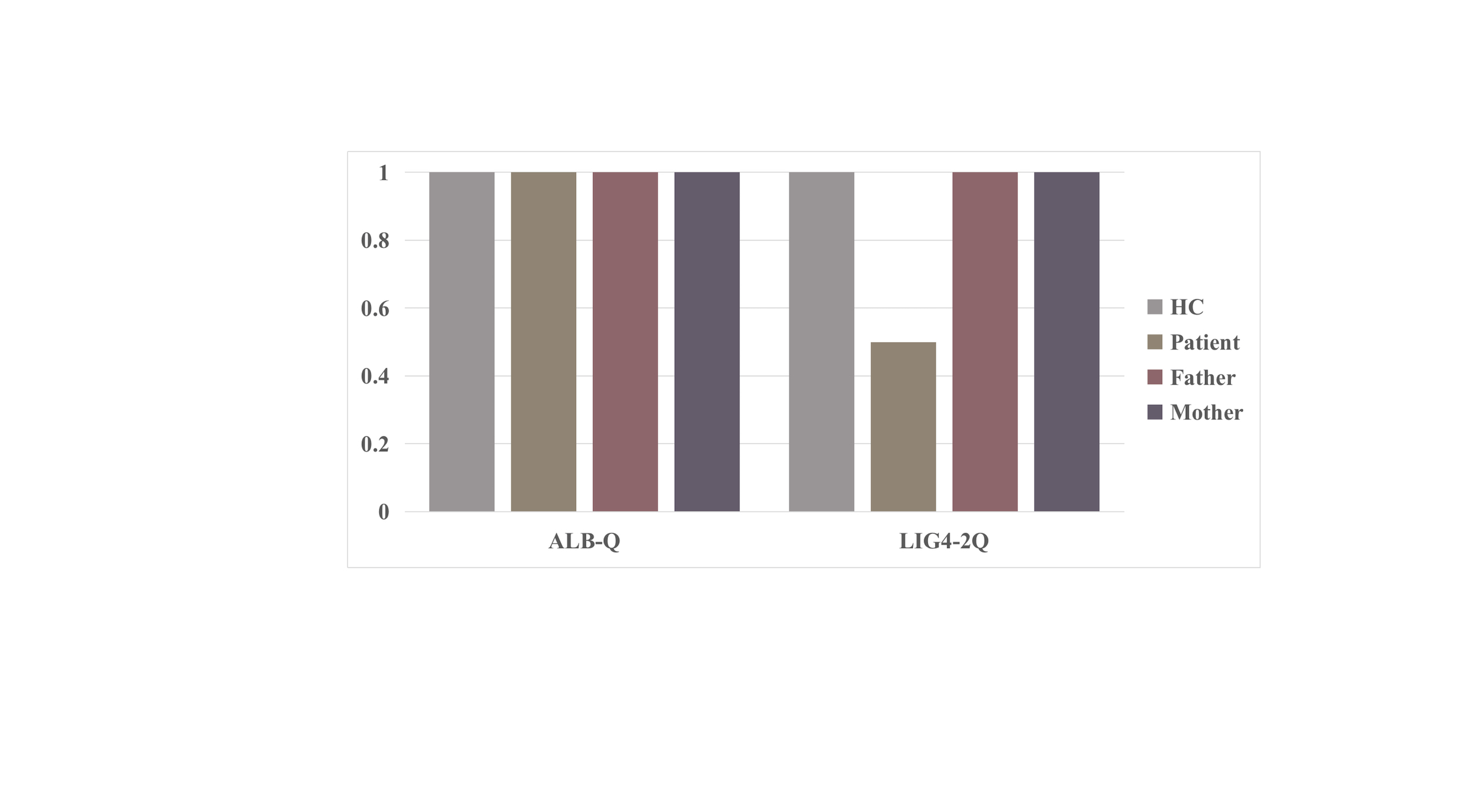


# Supplementary Figure: The copy number of exon 2 of the target gene LIG4 in P13 was detected using the ALB gene as the internal reference (fluorescence quantitative PCR).

**Supplementary Table.** SNPs used to reconstruct the haplotypes

| SNP | Alleles | Chromosome | MAF |
| --- | --- | --- | --- |
| rs9301287 | A>G, T | 13:108187603 (GRCh38) | A=0.466254/2335 (1000Genomes) |
| rs9559278 | G>A, T | 13:108192654 (GRCh38) | A=0.152356/763 (1000Genomes) |
| rs1931348 | G>A, C | 13:108193208 (GRCh38) | A=0.408546/2046 (1000Genomes) |
| rs1931349 | T>A, C, G | 13:108193250 (GRCh38) | T=0.354633/1776 (1000Genomes) |
| rs2391626 | G>A, C, T | 13:108195394 (GRCh38) | G=0.432508/2166 (1000Genomes) |
| rs915047 | G>A | 13:108198343 (GRCh38) | G=0.243011/1217 (1000Genomes) |
| rs9514825 | T>C | 13:108199928 (GRCh38) | T=0.432308/2165 (1000Genomes) |
| rs9520821 | C>G,T | 13:108200014 (GRCh38) | C=0.355032/1778 (1000Genomes) |
